# The Naloxone Project: Impact of Opioid Overdose Response Training on Medical Students’ Knowledge and Confidence

**DOI:** 10.7759/cureus.92279

**Published:** 2025-09-14

**Authors:** Putt Vithayaveroj, Emily Luo, Rithvic Jupudi, Nishanth Chalasani, Nathan Einhorn, Rebecca Lipscomb, Jennifer Costa

**Affiliations:** 1 Emergency Medicine, University of South Florida Morsani College of Medicine, Tampa, USA; 2 Anesthesiology, University of South Florida Morsani College of Medicine, Tampa, USA; 3 Family Medicine, University of South Florida Morsani College of Medicine, Tampa, USA; 4 Internal Medicine, University of South Florida Morsani College of Medicine, Tampa, USA; 5 Infectious Diseases, University of South Florida Morsani College of Medicine, Tampa, USA; 6 Biostatistics, University of South Florida Morsani College of Medicine, Tampa, USA

**Keywords:** harm reduction, naloxone education, opioid reversal, preclinical medical education, public health

## Abstract

Background

Early and practical education on naloxone, an opioid overdose reversal medication, is not commonly included in the curriculum of pre-clinical medical students. This study investigates how Opioid Overdose Response Training (OORT) improves opioid overdose recognition, naloxone administration, and opioid epidemic knowledge in pre-clinical medical students.

Objectives

This study aims to expand on the existing literature examining how OORT can supplement pre-clinical medical education regarding opioid reversal.

Methods

From 2022 to 2024, 187 pre-clinical medical students at the University of South Florida attended OORT with naloxone distribution. Students completed pre- and post-training surveys to subjectively rate their confidence and knowledge regarding opioid overdoses and naloxone administration. The Related Samples Wilcoxon Signed-Rank Test was used to assess differences in median responses. The Mann-Whitney U Test was used to compare pre-training responses between participants with and without prior OORT experience.

Results

After OORT, participants showed significant improvement in opioid overdose recognition (*p* < 0.001), naloxone administration (*p* < 0.001), and knowledge of the opioid epidemic (*p* < 0.001). The perceived importance of teaching OORT to the local community (*p* = 0.001) and teaching OORT to pre-clinical medical students (*p* = 0.012) remained high. Students with prior OORT experience (13.6%) reported significantly higher confidence in recognizing the signs of an opioid overdose (*p* < 0.001), administering naloxone (*p* < 0.001), and understanding the opioid epidemic (*p* < 0.001) compared to those without prior exposure. Additionally, they rated the importance of OORT for the community significantly higher (*p* = 0.047).

Conclusion

Pre-clinical medical students reported higher confidence and knowledge pertaining to the opioid epidemic and naloxone administration following OORT paired with naloxone distribution. Future research should investigate the long-term retention of OORT content as well as the added value of integrating naloxone distribution into training.

## Introduction

The opioid epidemic remains the leading cause of accidental death in the United States [1,2]. Between 2020 and 2021, deaths caused by synthetic opioids alone increased by 23% nationwide [3-5]. Naloxone, a commonly used opioid antagonist, reverses opioid toxicity through competitive binding to the mu-opioid receptor [6]. A 2017 modeling study found that life-saving naloxone distribution programs warranted substantial expansion in nearly every state [7].

In response to the opioid epidemic, Opioid Overdose Response Training (OORT) has become more widespread as a measure to reduce overdose risks among both laypeople and healthcare professionals. Several studies have found that OORT increases the knowledge and confidence of friends and family members of people who use opioids when naloxone administration is required [8]. Among healthcare professionals and medical students, OORT has shown some success in addressing attitudes toward opioid reversal techniques [9,10]. Even without naloxone distribution, OORT has been found to improve knowledge and preparedness among medical students, although it does not significantly change attitudes toward people with substance use disorders [11].

In medical education, simulation training provides students with deliberate practice in clinical decision-making [12]. Simulation-based skill application has been shown to improve knowledge among students learning Basic Life Support (BLS) [13]. While BLS training is mandatory in medical schools across the United States, OORT offers a more robust opioid and naloxone curriculum but has not yet been formally recognized as an essential component of pre-clinical education. Currently, opioid use disorder (OUD) education in medical school is primarily knowledge-based, lacking sufficient emphasis on curricular delivery, including faculty development and training resources. Although various methods for delivering OUD curricula have been described in published studies, student-developed curricula have been found to employ more diverse instructional approaches [14].

Our program employed a novel educational approach by integrating OORT with intranasal naloxone distribution. Each session provided trainees with naloxone as a salience-enhancing tool to reinforce learning among pre-clinical medical students. This study builds on existing literature by examining how pairing OORT with naloxone distribution improves overdose recognition, increases confidence in naloxone administration, and shapes perceptions of OORT's importance within both the community and the pre-clinical medical student population. By addressing a critical educational and practical gap, our program responds to the ongoing opioid epidemic and represents one of the first large-scale evaluations of student-led OORT with naloxone distribution among pre-clinical medical students.

## Materials and methods

Participants

A total of 187 pre-clinical medical students from the University of South Florida Morsani College of Medicine (USF MCOM) attended student-led OORT between April 2022 and March 2024 and were included in this study. Eligible participants were first- or second-year medical students who attended a one-hour, in-person training session. Recruitment was conducted via online advertisements in USF student group chats. Attendance at OORT was voluntary, and completion of pre- and post-training surveys was encouraged but optional. No incentives were given for survey completion.

Ethics statement

This retrospective analysis of a descriptive survey protocol was approved by the Institutional Review Board of USF (STUDY007570) and met the criteria for exemption from further review.

OORT

OORT sessions were led by USF medical students affiliated with a USF harm reduction student organization. The curriculum was adapted from a training program developed by the Florida Department of Children and Families. The one-hour, in-person course included content on the history of the opioid crisis, signs and symptoms of opioid overdose, the mechanism of action and administration technique for naloxone, and legal protections for individuals who report an overdose. Demonstration naloxone was used during the training, and unopened naloxone kits were distributed to interested students at the conclusion of each session, regardless of survey completion.

Pre- and post-training surveys

Before and after each OORT session, participants were asked to complete an online survey accessed via QR code. The pre- and post-training surveys were administered immediately before and after OORT. Responses were rated on a 5-point Likert scale, where ‘1’ indicated the lowest rating and ‘5’ the highest. The first three survey questions (Q1-Q3) asked participants to self-assess their confidence in understanding the opioid epidemic, recognizing opioid overdoses, and administering naloxone. These questions were consistent across all cohorts. Two additional questions assessed the perceived importance of OORT for pre-clinical medical students and for the broader community. Of note, Q5 regarding the importance of OORT for pre-clinical medical students was asked only to the first cohort (n=49), while Q4 regarding community education was asked to all subsequent participants (n=138), which explains the disparity in survey responses. A supplemental question inquired about any prior naloxone training experience. The same set of questions was used in both the pre- and post-training surveys.

Statistical analysis

Survey data were collected via Google Forms (Google LLC, Mountain View, CA, USA) between 2022 and 2024. Responses were de-identified and analyzed by a statistician from the USF Research, Innovation & Scholarly Endeavors (RISE) program. Due to changes in the survey rating scale between the 2022 and 2023 forms, responses of ‘0’ and ‘1’ were combined for analysis.

The Related Samples Wilcoxon Signed Rank Test was used to determine whether there was a significant median difference between pre- and post-training confidence in opioid overdose recognition. For questions where the distribution of differences between paired observations was neither normal nor symmetrical, the Sign Test was applied. The Mann-Whitney U Test was used to compare pre-training responses between participants with and without prior naloxone training. P-values less than 0.05 were considered statistically significant. All statistical analyses were performed using IBM SPSS Statistics for Windows, versions 29 and 30 (IBM Corp., Armonk, NY, USA).

## Results

Between 2022 and 2024, a total of 187 pre-clinical medical students participated in student-led OORT sessions and completed pre- and post-training surveys. For Q1-Q3, which assessed confidence in opioid-related knowledge and skills, the overall response rates were 90.4% (pre-training) and 84.0% (post-training) (Table 1). Q5, which assessed the perceived importance of OORT for pre-clinical medical students, was asked only in the first cohort (n=49), with response rates of 85.7% pre-training and 83.7% post-training. Q4, which focused on the importance of OORT for the broader community, was asked of all subsequent participants (n=138), with pre- and post-training response rates of 92.0% and 94.1%, respectively. Across all cohorts, 22 participants (11.8%) reported previous naloxone training. Among the 169 participants who completed the pre-training survey, 23 (13.6%) reported having attended a prior OORT session.

**Table 1:** Comparison of pre- and post-training survey responses in pre-clinical medical students. For Q1, the Wilcoxon Sign Rank Test was used. For questions Q2-Q5, the Sign Test was used. n: number; IQR: interquartile range; SD: standard deviation; Q1: question 1; Q2: question 2; Q3: question 3; Q4: question 4; Q5: question 5.

Question		Pre-training			Post-training			p-value
		n	Median (IQR)	Mean ± SD	n	Median (IQR)	Mean ± SD	
Q1	How confident are you at recognizing signs of an opioid overdose?	169	2 (1-3)	2.31 ± 1.2	157	4 (4-5)	4.23 ± 0.81	<0.001
Q2	How confident are you at administering naloxone?	169	1 (1-2.5)	1.78 ± 1.17	157	5 (4-5)	4.55 ± 0.75	<0.001
Q3	How much do you know about the current opioid epidemic?	169	3 (3-4)	3.16 ± 1.02	157	4 (4-5)	4.13 ± 0.75	<0.001
Q4	How important do you think it is for the local community to learn about the opioid epidemic and naloxone administration?	127	5 (4-5)	4.65 ± 0.69	116	5 (5-5)	4.88 ± 0.33	0.001
Q5	How important do you think it is for preclinical medical students to learn about the opioid epidemic and naloxone administration?	42	4 (3-5)	3.88 ± 1.17	41	4 (4-5)	4.29 ± 0.84	0.012

**Figure 1:**
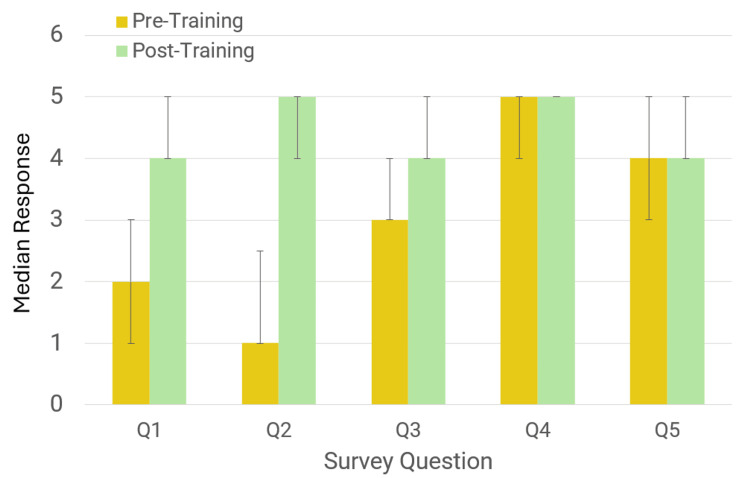
Median scores (with IQR) of pre- and post-training survey responses in pre-clinical medical students. IQR: interquartile range; Q1: How confident are you at recognizing signs of an opioid overdose?; Q2: How confident are you at administering naloxone?; Q3: How much do you know about the current opioid epidemic?; Q4: How important do you think it is for the local community to learn about the opioid epidemic and naloxone administration?; Q5: How important do you think it is for preclinical medical students to learn about the opioid epidemic and naloxone administration?

Comparisons of median and mean responses to the survey questions are presented in Table 1 and Figure 1. Participants demonstrated a statistically significant overall increase in median scores following OORT. Confidence in recognizing the signs of opioid overdose significantly improved (p < 0.001), with the median score increasing from 2 (IQR 1-3) to 4 (IQR 3-5), and the mean rising from 2.31 (SD 1.2) to 4.23 (SD 0.81), representing a two-fold increase in reported confidence. Confidence in naloxone administration also showed a statistically significant increase (p < 0.001), with the median rising from 1 (IQR 1-2.5) to 5 (IQR 4-5), and the mean increasing from 1.78 (SD 1.17) to 4.55 (SD 0.75). Participants’ knowledge of the opioid epidemic improved significantly as well (p < 0.001), with the median score increasing from 3 (IQR 3-4) to 4 (IQR 4-5), and the mean rising from 3.16 (SD 1.02) to 4.13 (SD 0.75).

Questions regarding the importance of providing community education and incorporating training into the first-year medical curriculum received high baseline scores and still showed improvement in post-training responses. The perceived importance of community education remained high in both pre- and post-training surveys, with a median score of 5 (p = 0.001) and a slight increase in mean from 4.65 (SD 0.69) to 4.88 (SD 0.33). Similarly, the importance of integrating such training into medical curricula was rated highly in both surveys, with the median remaining at 4 (p = 0.012) and the mean increasing from 3.88 (SD 1.37) to 4.29 (SD 0.84).

When comparing pre-training survey responses between participants with prior naloxone training and those without (Table 2), those with previous training demonstrated significantly higher scores on multiple items, including confidence in recognizing signs of an opioid overdose (p < 0.001), confidence in administering naloxone (p < 0.001), knowledge about the current opioid epidemic (p < 0.001), and perceived importance of OORT for the local community (p = 0.047). The only exception was the perceived importance of OORT for pre-clinical medical students, which was not statistically significant (p = 0.098).

**Table 2:** Comparison of pre-training survey questions between prior naloxone training groups (no/yes). For Q1-Q5, the Mann Whitney U Test was used. n: number; IQR: interquartile range; Q1: question 1; Q2: question 2; Q3: question 3; Q4: question 4; Q5: question 5.

Question		Prior naloxone training?		p-value
		No (n=146)	Yes (n=23)	
		Median (IQR)	Median (IQR)	
Q1	How confident are you at recognizing signs of an opioid overdose?	2 (1-3)	4 (3-5)	<0.001
Q2	How confident are you at administering naloxone?	1 (1-2)	3 (3-5)	<0.001
Q3	How much do you know about the current opioid epidemic?	3 (3-4)	4 (3-4)	<0.001
Q4	How important do you think it is for the local community to learn about the opioid epidemic and naloxone administration?	5 (4-5)	5 (5-5)	0.047
Q5	How important do you think it is for pre-clinical medical students to learn about the opioid epidemic and naloxone administration?	4 (3-5)	2.5 (2-3)	0.098

## Discussion

Pre-clinical medical students showed a statistically significant increase in confidence in recognizing the signs of an opioid overdose, administering intranasal naloxone, and understanding the opioid epidemic after receiving OORT. The disparity between pre- and post-training confidence and knowledge may reflect curricular gaps in socially relevant medical education, often not addressed until later stages of training. Furthermore, students’ perceptions of the importance of opioid epidemic education and naloxone training, both in the community and for pre-clinical students, improved. This indicates strong support for the relevance of this content among medical trainees.

Although changes in perception were statistically significant, median scores remained consistent pre- and post-training, suggesting that students may have had an existing baseline interest in these topics prior to OORT. These findings highlight an opportunity to enhance pre-clinical education through experiential learning that addresses contemporary public health issues. A secondary observation was that students with prior naloxone training reported significantly higher confidence in recognizing overdose signs, administering naloxone, and understanding the opioid crisis. This suggests that OORT may lead to long-term retention of knowledge and confidence.

Overall, our results align with previous studies that have demonstrated increased knowledge and confidence among healthcare professionals and medical students following OORT [9-11]. However, previously studied OORTs did not include naloxone distribution, a key component of our training. Future studies may compare the benefits of OORT sessions that include naloxone distribution with those that do not. Interprofessional OORTs have yielded similar outcomes among nurses, physician assistants, and pharmacy students in the form of knowledge, confidence, and collaboration scores [9]. While our findings support the positive impact of repeated OORT exposure, the timeline between initial training and sustained confidence remains underexplored. One study found that initial confidence in overdose response among first-year medical students regressed after three months [10]. However, in a follow-up study involving the same cohort, half of the students received a brief “booster” video at 2.5 months. At the three-month follow-up, the booster group exhibited decreased stigma and increased willingness to respond to overdoses compared to their peers who did not receive a second intervention [15]. These findings suggest that early training combined with follow-up reinforcement may enhance and sustain the benefits of OORT.

Additionally, other studies suggest that OORT leads to better long-term retention compared to standard medical education alone, with effects persisting up to six months post-training [16]. This corroborates our finding that students with prior OORT experience reported greater confidence and knowledge, further supporting the case for integrating OORT into the national pre-clinical medical curriculum. These insights underscore the need for future research to evaluate the long-term impact of pre-clinical OORT, particularly by comparing confidence levels among clinical students who did or did not receive OORT during their pre-clinical years.

Our curriculum distinguished itself from other similar training programs by partnering with local harm reduction organizations and integrating free naloxone distribution to increase the practicability and salience of OORT for participating pre-clinical medical students. Future research could explore whether pairing OORT with naloxone distribution improves retention compared to training alone, a question that current literature has yet to adequately address. Collaborations between health professional programs and harm reduction organizations, government agencies, or hospital pharmacies may facilitate wider implementation of this approach. One example is a study in which medical students partnered with their hospital pharmacy to distribute naloxone via Health Insurance Portability and Accountability Act (HIPAA)-secure electronic prescriptions processed daily [17]. This demonstrates the feasibility of diverse distribution models to ensure that medical students are equipped with naloxone and prepared to intervene in opioid overdose situations. Future programs may benefit from deeper collaborations with local organizations, helping medical students gain awareness of community-based resources.

Strengths and limitations

One strength of this study was the collaboration between student educators and the Department of Children and Families in developing the OORT curriculum. All student instructors completed a formal training course through the Department, and our materials were adapted from their official curriculum, ensuring the use of up-to-date and evidence-based content. In addition, all sessions were led by the same group of student instructors, promoting consistency in both delivery and content across trainings. The inclusion of naloxone distribution added a practical, real-world component that is often missing in other student-led OORT initiatives.

There were a few limitations to our study. First, the generalizability of our findings was limited by the study population, which consisted solely of voluntary participants enrolled at USF MCOM. Second, the surveys used in this study were not based on previously validated instruments. Additionally, one iteration of the survey in 2022 used ‘0’ as the minimum response value, while other surveys used ‘1’. To maintain consistency, responses of ‘0’ were converted to ‘1’; while we believe this had minimal impact, it introduces a potential source of error in interpreting lower-end responses. Large discrepancies in participation data were caused by the variability of Q4 and Q5 on our training surveys, as Q5 was limited to the first cohort of participants while Q4 was used for all other participants. This change was primarily due to the expansion of our training audience to include community groups outside USF MCOM. Finally, the number of responses varied between pre- and post-training surveys due to incomplete participation, which may have reduced the statistical power of findings, particularly regarding perceptions of the importance of OORT in pre-clinical education.

## Conclusions

Pre-clinical medical students post-OORT demonstrated a statistically significant improvement in their confidence in recognizing opioid overdoses, administering intranasal naloxone, and understanding the broader context of the opioid epidemic. These are essential skills for any future healthcare professional, particularly given the growing impact of opioid use and addiction on public health. Our findings support the integration of OORT into pre-clinical training for medical students nationwide. Future research should examine long-term knowledge retention, comparing outcomes for students who received OORT paired with naloxone distribution versus OORT alone. Additional studies might also evaluate the long-term confidence and knowledge levels of clinical medical students who received pre-clinical OORT training compared to those who did not. These efforts could help solidify the role of OORT in shaping compassionate, knowledgeable, and prepared future physicians.
